# Midlife Intakes of the Isoflavone Genistein and Soy and the Risk of Late-life Cognitive Impairment: The JPHC Saku Mental Health Study

**DOI:** 10.2188/jea.JE20210199

**Published:** 2023-07-05

**Authors:** Thomas Svensson, Norie Sawada, Masaru Mimura, Shoko Nozaki, Ryo Shikimoto, Shoichiro Tsugane

**Affiliations:** 1 Epidemiology and Prevention Group, Center for Public Health Sciences, National Cancer Center, Tokyo, Japan; 2 Department of Neuropsychiatry, Keio University School of Medicine, Tokyo, Japan; 3 Precision Health, Department of Bioengineering, Graduate School of Engineering, The University of Tokyo, Tokyo, Japan; 4 Department of Clinical Sciences, Lund University, Skåne University Hospital, Malmö, Sweden; 5 School of Health Innovation, Kanagawa University of Human Services, Kanagawa, Japan

**Keywords:** dementia, general population cohort, isoflavones, late-life, midlife, mild cognitive impairment, soy foods

## Abstract

**Background:**

The number of people with cognitive impairment, including dementia, in the world is steadily increasing. Although the consumption of isoflavones and soy is associated with a reduced risk of cardiovascular disease, it might also be associated with cognitive impairment. The low number of studies investigating the association between soy/isoflavone intake and cognitive function warrant additional research.

**Methods:**

The Japan Public Health Center-based prospective (JPHC) Study is a large population-based cohort. Midlife dietary intake of soy and the isoflavone genistein was assessed on two occasions: in the years 1995 and 2000. In 2014–2015, 1,299 participants from Nagano prefecture completed a mental health screening. Of these, a total of 1,036 participants were included in analyses. Logistic regression was used to determine Odds Ratios (OR) and 95% Confidence Intervals (CI) for the association between midlife energy-adjusted genistein and soy food intake and cognitive impairment.

**Results:**

There were 392 cases of cognitive impairment (346 cases of MCI and 46 cases of dementia). Compared to the lowest dietary quartile of energy-adjusted genistein intake, the highest quartile was significantly associated with cognitive impairment (OR = 1.51; 95% CI, 1.02–2.24; *P* for trend = 0.03) in the final multivariable analysis.

**Conclusion:**

High midlife intake of the isoflavone genistein is associated with late-life cognitive impairment.

## INTRODUCTION

The number of people with dementia in the world is expected to more than double by 2050.^1^ One third of dementia cases could be attributable to modifiable risk factors.^2^ It is therefore crucial to identify any factors that could aid in the early intervention and prevention of cognitive decline, including mild cognitive impairment (MCI), as individuals with MCI progress to dementia at higher rates than those who are cognitively healthy.^3^

Soy and isoflavones, phytoestrogens for which the predominant food source in Asia is soybean, have garnered attention for their beneficial health effects. The consumption of isoflavones is associated with a reduced risk of cerebral and myocardial infarction among women,^4^ and fermented soy products may protect against high blood pressure^5^ and cardiovascular disease mortality.^6^ Contrary to the evidence supporting a cardioprotective effect of dietary isoflavone and soy intake, the findings from observational studies indicate that tofu consumption is inversely associated with memory,^7^ cognitive performance,^8^ and cognitive function.^9^ The only soy food with a reportedly positive effect on cognition is tempeh, a fermented soy product which in one study offset the negative effect of tofu.^7^

Observational studies have considered the habitual consumption of soy/isoflavones concomitantly with the assessment of cognitive function, thereby increasing the risk of introducing reverse causation bias. The long-term effects of isoflavone intake on cognitive impairment have, to the best of our knowledge, been investigated in only two studies, one of which reported a protective effect for women only^10^ and another which reported no association.^11^ Additional studies investigating the association between midlife soy and isoflavone intake and late-life cognitive impairment are therefore warranted.

The purpose of the present study was to investigate, in a Japanese general population cohort, the associations of energy-adjusted midlife dietary intake of the isoflavone genistein and soy with cognitive impairment, defined as a composite of late-life MCI and dementia. In accordance with available research, we hypothesize that intake of fermented soy products, as well as high genistein intake, may be inversely associated with cognitive impairment.

## METHODS

### Study population

**Figure 1.**
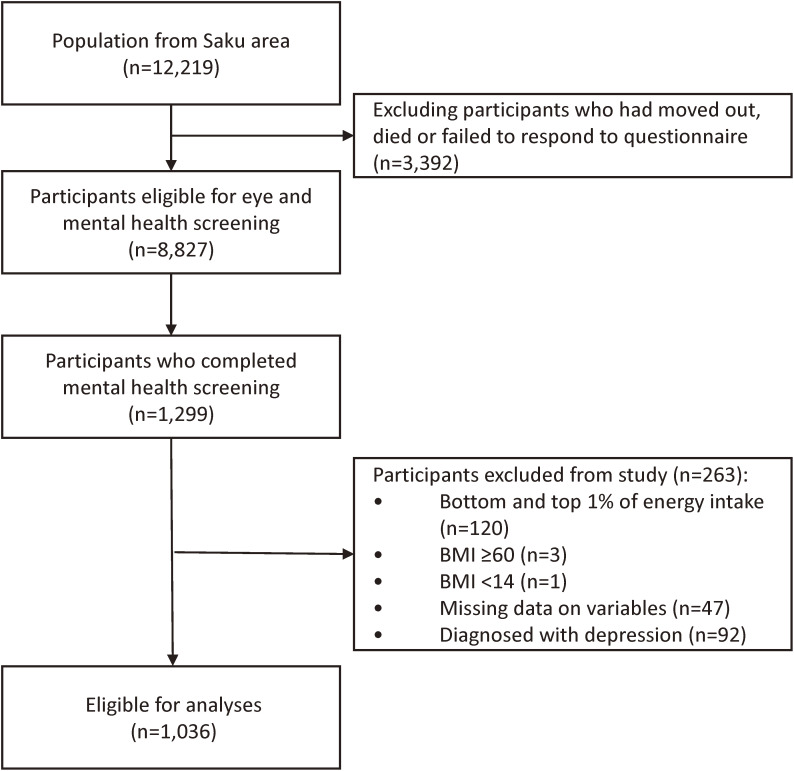
Flowchart for the inclusion and exclusion of study participants

The Japan Public Health Center-based prospective (JPHC) Study is a large population-based cohort which was started in 1990 and was conducted in two cohorts; cohort I was initiated in 1990 and cohort II in 1993. The study design has been described in detail elsewhere.^12^ JPHC Study participants were identified using population registries maintained by the local municipalities in 11 public health center (PHC) areas. There were 140,420 individuals identified in the JPHC study at baseline. The study population of the present study were the 12,219 JPHC study participants who in 1990 were between the ages of 40 and 59 and residents of Saku PHC area in Nagano Prefecture (Figure 1). In 2014–2015, 8,827 individuals were considered eligible for an invitation to participate in a mental health screening, following the exclusion of those who had moved out of Saku PHC area, had died, or had not responded to the JPHC Study baseline questionnaire. A total of 1,299 individuals completed the screening.

Following the exclusion of those with an average energy-adjusted intake (in kilocalories [kcal]) in the top (>4,279.4 kcal [men] and >4,418.6 kcal [women]) or bottom (<1,284.33 kcal [men] and <1,033.1 kcal [women]) percentile (*n* = 120), body mass index (BMI) values <14 kg/m^2^ (indicative of severe underweight and possibility of concomitant disease) or >60 kg/m^2^ (indicative of a high possibility of unreasonable values) (*n* = 4), missing data for any of the adjusting variables (*n* = 47), or those who had been diagnosed with depression at the mental health screening (*n* = 92), there were 1,036 participants eligible for analyses in the present study. Of the 1,036 eligible participants, 46 individuals were diagnosed with dementia and 346 individuals with MCI at the time of screening.

JPHC study participants were followed-up on three occasions with detailed questionnaires on diet, socioeconomic, and lifestyle factors. The present study used information from all three surveys: baseline (1990–1992), 5-year follow-up (1995), and 10-year follow-up (2000).

All participants provided written informed consent to take part in the mental health screening in 2014–2015, and the study has been approved by the Institutional Review Board of the National Cancer Center (approval number: 2013-096).

### Soy and isoflavone intake

Dietary intake of soy and genistein was assessed through the same self-administered 147-item food frequency questionnaire (FFQ) at both the 5-year and 10-year follow-up surveys. For each food item, the pre-specified food consumption frequencies and portion sizes were multiplied to calculate daily food consumption of the respective food item. The daily dietary intake of nutrients was calculated based on the 5^th^ Revised and Enlarged edition of the Standard Tables of Food Composition in Japan.^13^

Eight soy food items were included in the FFQ: miso soup, soymilk, tofu for miso soup, tofu for other dishes, yushidofu (pre-drained tofu), koyadofu (freeze-dried tofu), aburaage (deep-fried tofu), and natto (fermented soybeans). Detailed consumption frequencies and amount depended on the specific soy food item: miso soup (almost never, 1–3 days/month, 1–2 days/week, 3–4 days/week, 5–6 days/week, or daily, and the number of 150 mL bowls per day: <1, 1, 2, 3, 4, 5, 6, 7–9 or >9), soymilk (almost never, 1–3 times/month, 1–2 times/week, 3–4 times/week, 5–6 times/week, and the number of 200 mL glasses per day: 1, 2–3, 4–6, 7–9 or >9), and remaining soy food items (frequencies: almost never, 1–3 times/month, 1–2 times/week, 3–4 times/week, 5–6 times/week, 1 time/day, 2–3 times/day, 4–6 times/day and ≥7 times/day, and portion sizes: small [50% smaller than standard], medium [same as standard], and large [50% larger than standard]).

The daily intake of genistein and daidzein were based on a specially developed food composition table for isoflavones in Japanese foods.^14^^,^^15^ Due to the high correlation between genistein and daidzein, genistein was chosen as representative of isoflavone intake in the present study. The FFQ-derived energy-adjusted genistein intake has been validated in a subsample with consecutive 14- or 28-day dietary records in which Spearman’s correlation coefficients between the FFQ and the dietary records were 0.65 for men and 0.55 for women.^16^ Spearman’s correlation coefficients for the reproducibility of energy-adjusted genistein intake between the two questionnaires assessed 1 year apart was 0.75 for men and 0.69 for women.^16^

For the purpose of the present study, the sex-specific average energy-adjusted intake of soy foods was calculated based on the 5-year and 10-year FFQs. Food and nutrient intake was log-transformed and adjusted for total energy intake using the residual method.^17^

There were six main exposure groups: overall genistein intake, overall soy food, tofu, miso, natto, and fermented soy (miso and natto combined). For each of the groups, quartiles of energy-adjusted intake were constructed based on the cut-off points for participants who were free from MCI and dementia at the time of the mental health screening.

### Mild cognitive impairment and dementia

Cognitive function was assessed by experienced neuropsychologists using the Mini-Mental State Examination (MMSE),^18^ the Wechsler Memory Scale Revised (WMS-R) logical memory I/II subtest,^19^ the clock drawing test,^20^ and the Clinical Dementia Rating (CDR) Scale.^21^ Participants’ cognitive function was subsequently categorized in accordance with criteria used in the Japanese Alzheimer’s Disease Neuroimaging Initiative (J-ADNI) project,^22^^,^^23^ where MCI was defined as amnestic MCI as originally presented by Petersen et al.^24^ Memory impairment was assessed based on an education-adjusted score below the cut-off level on the WMS-R Logical Memory II test (education for 0–9 years was 2, for 10–15 years was 4, and for >15 years was 8). The MMSE cut-off point for dementia was set at 23. Depressive symptoms were assessed using the Center for Epidemiologic Studies Depression Scale (CES-D),^25^ and the Patient Health Questionnaire-9 (PHQ-9).^26^ A trained psychiatrist combined the neuropsychological assessment with a clinical interview to determine the final diagnosis in accordance with the Diagnostic and Statistical Manual of Mental Disorders, 4th. Edition Text Revision (DSM-IV-TR) criteria.^27^

### Covariates

Education was from the baseline questionnaire and categorized as junior high school, high school, or college/vocational school/other; alcohol consumption (binarized as <150 or ≥150 g ethanol per week), smoking status (non-smoker, past smoker, or current smoker), body mass index (BMI in kg/m^2^), and physical activity (defined as metabolic equivalents) were from the 5-year follow-up questionnaire; age, prescribed medication (defined as having taken any medications for psychiatric or neurological conditions), self-reported history of diabetes mellitus (yes/no), and self-reported history of stroke (yes/no) were obtained at the time of the mental health screening; All food and nutrient intake (ie, folate, fish, meat, vegetables, fruits, and sodium) were calculated as sex-specific average energy-adjusted intake based on the 5-year and 10-year FFQs. Intake was log-transformed and adjusted for total energy intake using the residual method.^17^

### Statistical analyses

Differences in baseline characteristics between quartiles of energy-adjusted genistein intake were determined using the Chi-square test for categorical variables and analysis of variance for continuous variables.

Logistic regression models were used to determine odds ratios (ORs) and 95% confidence intervals (CIs) for the association between each of the genistein and soy food groups and cognitive impairment. Model 1 was adjusted for age (continuous), sex, education, and energy-adjusted folate intake. Model 2 was additionally adjusted for alcohol consumption, smoking status, BMI (continuous), and physical activity. Model 3 was further adjusted for use of prescribed medication, a self-reported history of diabetes mellitus, and a self-reported history of stroke. Model 4 was additionally adjusted for energy-adjusted intake of fish, meat, vegetables, fruits, and sodium. Missing data was accounted for by listwise deletion. Sensitivity analyses were stratified by sex.

Statistical analyses were performed using SAS (SAS software version 9.4; SAS institute Inc., Cary, NC, USA). *P*-values were two-tailed and considered significant if *P* < 0.05.

## RESULTS

Baseline characteristics for complete cases according to quartiles of energy-adjusted genistein intake show that those in the top quartile were older, had the highest intake of folate, fish, fruits, and sodium, and consumed the least amount of meat (Table 1). The mean age and energy-adjusted genistein intake of those without cognitive impairment at the time of screening was 72.3 (standard deviation [SD], 5.4) years, and 26.5 (SD, 13.6) mg/day, respectively.

**Table 1.** Baseline characteristics according to quartiles of isoflavone intake for all eligible participants (*n* = 1,036)

Characteristics	Quartile intake of isoflavone (Median intake mg/day)				*P* value^a^

	Quartile 1 (13.7)	Quartile 2 (21.1)	Quartile 3 (28.5)	Quartile 4 (40.3)	
**Proportion of participants, %**	23.9	25.7	22.7	27.7	
**Age,^b^ years, mean (SD)**	72.3 (5.4)	73.1 (5.6)	73.2 (5.5)	73.9 (5.6)	0.01
**Men (%)**	39.5	44.4	40.0	42.9	0.64
**Education, %**					0.18
Junior high school	29.0	24.4	31.5	35.5	
High school	52.4	55.6	50.6	49.1	
College/vocational school, University or Other	18.6	19.9	17.9	15.3	
**Alcohol consumption, %**					0.42
<150 g ethanol per week	79.0	74.8	74.5	79.1	
≥150 g ethanol per week	21.0	25.2	25.5	20.9	
**Smoking status, %**					0.22
Non-smoker	69.0	69.9	74.0	72.5	
Past smoker	11.7	9.0	8.5	11.5	
Current smoker <20 cigarettes/day	6.9	6.8	4.3	8.4	
Current smoker ≥20 cigarettes/day	12.5	14.3	13.2	7.7	
**Body Mass Index, kg/m^2^, mean (SD)**	23.2 (3.1)	23.4 (2.6)	23.4 (2.6)	23.6 (2.7)	0.54
**Physical activity, MET-h/day, mean (SD)**	37.5 (9.6)	38.2 (10.0)	37.3 (9.5)	37.7 (10.0)	0.75
**History of diabetes mellitus, %**	8.1	8.3	10.2	10.1	0.75
**History of stroke, %**	5.2	4.1	4.3	3.5	0.80
**Prescribed medication, %**	10.5	9.0	8.1	12.5	0.35
**Energy-adjusted mean dietary intake**					
**Folate, µg/day, mean (SD)**	448.0 (135.9)	477.4 (129.6)	530.3 (126.8)	555.2 (125.6)	<0.001
**Meat, g/day, mean (SD)**	72.2 (39.7)	71.9 (34.6)	72.9 (40.7)	63.3 (31.1)	0.002
**Fish, g/day, mean (SD)**	97.6 (47.0)	105.2 (50.3)	103.7 (41.2)	108.1 (44.7)	0.07
**Vegetables, g/day, mean (SD)**	287.8 (160.2)	321.4 (161.1)	355.8 (144.3)	347.3 (151.1)	<0.001
**Fruits, g/day, mean (SD)**	256.6 (167.7)	255.0 (157.3)	268.5 (154.2)	289.2 (150.0)	0.04
**Sodium, g/day, mean (SD)**	5.2 (1.4)	5.8 (1.3)	6.1 (1.4)	6.2 (1.4)	<0.001

SD, standard deviation.

^a^Analysis of Variance for continuous variables; Chi-square test for categorical variables.

^b^Age at the time of mental health screening.

### Cognitive impairment

At the mental health screening, 392 participants were diagnosed with cognitive impairment, defined as MCI or dementia. The mean age and energy-adjusted genistein intake of those diagnosed with cognitive impairment at the time of screening was 74.5 (SD, 5.6) years, and 29.2 (SD, 16.2) mg/day, respectively.

Compared to those with the lowest energy-adjusted intake (quartile 1) of genistein in model 1, individuals with the highest intake (quartile 4) had significantly higher odds of cognitive impairment (OR 1.58; 95% CI, 1.08–2.31) (Table 2). The association was only slightly attenuated by the addition of covariates and remained significant also in the fully adjusted model (Model 4: OR 1.51; 95% CI, 1.02–2.24). There was a significant linear trend for the association between genistein intake and cognitive impairment (*P* = 0.03).

**Table 2.** Odds ratios and their confidence intervals of cognitive impairment according to energy-adjusted intake of isoflavone, soy food, tofu, miso soup, and fermented soy products

	Quartile intake				*P* for trend

	Quartile 1 (Low)	Quartile 2	Quartile 3	Quartile 4 (High)	
**Isoflavone**					
Number (Events)	248 (84)	266 (95)	235 (86)	287 (127)	
mg/day, median [range]	13.7 [4.2–17.2]	21.1 [17.2–24.8]	28.5 [24.7–32.6]	40.3 [32.6–161.3]	
Model 1^a^ OR (95% CI)	Reference	1.06 (0.73–1.54)	1.17 (0.79–1.74)	1.58^*^ **(1.08–2.31)**	0.01
Model 2^b^ OR (95% CI)	Reference	1.03 (0.70–1.50)	1.18 (0.79–1.76)	1.57^*^ **(1.07–2.31)**	0.01
Model 3^c^ OR (95% CI)	Reference	1.04 (0.71–1.52)	1.19 (0.80–1.78)	1.56^*^ **(1.06–2.30)**	0.02
Model 4^d^ OR (95% CI)	Reference	1.01 (0.69–1.48)	1.15 (0.77–1.72)	1.51^*^ **(1.02–2.24)**	0.03

**Soy food**					
Number (Events)	260 (97)	225 (67)	250 (88)	301 (140)	
g/day, median [range]	38.6 [7.9–50.9]	58.5 [47.8–70.1]	78.4 [65.5–92.9]	115.8 [87.0–966.4]	
Model 1^a^ OR (95% CI)	Reference	0.72 (0.49–1.07)	0.89 (0.61–1.31)	1.48^*^ **(1.02–2.14)**	0.01
Model 2^b^ OR (95% CI)	Reference	0.72 (0.48–1.06)	0.88 (0.60–1.30)	1.48^*^ **(1.02–2.14)**	0.02
Model 3^c^ OR (95% CI)	Reference	0.71 (0.48–1.06)	0.87 (0.59–1.28)	1.46^*^ **(1.01–2.13)**	0.02
Model 4^d^ OR (95% CI)	Reference	0.68 (0.46–1.02)	0.82 (0.55–1.21)	1.41 (0.97–2.06)	0.03

**Tofu**					
Number (Events)	248 (86)	247 (82)	287 (124)	254 (100)	
g/day, median [range]	11.3 [−0.27–16.8]	21.6 [15.9–27.2]	32.4 [26.1–41.7]	55.2 [40.0–186.9]	
Model 1^a^ OR (95% CI)	Reference	0.95 (0.65–1.39)	1.41 (0.98–2.02)	1.16 (0.79–1.68)	0.16
Model 2^b^ OR (95% CI)	Reference	0.96 (0.66–1.42)	1.40 (0.97–2.01)	1.15 (0.79–1.68)	0.18
Model 3^c^ OR (95% CI)	Reference	0.95 (0.65–1.41)	1.39 (0.96–2.01)	1.15 (0.79–1.68)	0.18
Model 4^d^ OR (95% CI)	Reference	0.95 (0.64–1.40)	1.33 (0.92–1.93)	1.13 (0.77–1.67)	0.24

**Miso soup**					
Number (Events)	261 (100)	245 (82)	250 (90)	280 (120)	
mL/day, median [range]	36.1 [−0.12–76.2]	87.8 [58.7–135.8]	141.4 [105.8–187.9]	202.8 [144.8–639.2]	
Model 1^a^ OR (95% CI)	Reference	0.79 (0.54–1.14)	0.83 (0.57–1.20)	1.17 (0.82–1.67)	0.33
Model 2^b^ OR (95% CI)	Reference	0.78 (0.54–1.14)	0.81 (0.56–1.18)	1.15 (0.80–1.65)	0.40
Model 3^c^ OR (95% CI)	Reference	0.79 (0.54–1.15)	0.82 (0.56–1.19)	1.19 (0.83–1.71)	0.31
Model 4^d^ OR (95% CI)	Reference	0.78 (0.53–1.15)	0.80 (0.54–1.19)	1.19 (0.81–1.75)	0.38

**Natto**					
Number (Events)	257 (97)	254 (91)	247 (89)	278 (115)	
g/day, median [range]	3.5 [−0.46–6.3]	8.1 [5.6–10.7]	13.2 [9.5–18.3]	23.9 [16.2–83.1]	
Model 1^a^ OR (95% CI)	Reference	0.94 (0.65–1.36)	0.93 (0.64–1.35)	1.18 (0.81–1.70)	0.39
Model 2^b^ OR (95% CI)	Reference	0.94 (0.65–1.37)	0.93 (0.64–1.36)	1.20 (0.83–1.74)	0.34
Model 3^c^ OR (95% CI)	Reference	0.92 (0.63–1.34)	0.93 (0.63–1.36)	1.20 (0.83–1.75)	0.32
Model 4^d^ OR (95% CI)	Reference	0.91 (0.62–1.34)	0.90 (0.61–1.32)	1.18 (0.81–1.72)	0.41

**Fermented soy (Natto+Miso)**					
Number (Events)	245 (83)	255 (95)	260 (96)	276 (118)	
g/day, median [range]	46.7 [3.6–86.0]	97.4 [70.3–146.7]	155.5 [120.0–199.3]	216.0 [163.2–647.5]	
Model 1^a^ OR (95% CI)	Reference	1.14 (0.78–1.66)	1.06 (0.73–1.54)	1.41 (0.97–2.03)	0.10
Model 2^b^ OR (95% CI)	Reference	1.11 (0.76–1.62)	1.04 (0.71–1.51)	1.37 (0.94–1.98)	0.14
Model 3^c^ OR (95% CI)	Reference	1.11 (0.76–1.63)	1.04 (0.71–1.52)	1.41 (0.97–2.05)	0.10
Model 4^d^ OR (95% CI)	Reference	1.09 (0.74–1.61)	1.02 (0.69–1.52)	1.38 (0.93–2.07)	0.16

CI, confidence interval; OR, odds ratio.

^*^P < 0.05;

^a^Model 1 is adjusted for age, gender, education and energy-adjusted folate intake;

^b^Model 2 is additionally adjusted for alcohol consumption, smoking, body mass index, and physical activity;

^c^Model 3 is additionally adjusted for history of diabetes mellitus, history of stroke, and use of prescribed medications;

^d^Model 4 is additionally adjusted for energy-adjusted intake of fish, meat, vegetables, fruits, and sodium.

Compared to the lowest soy food intake (quartile 1), the highest soy food intake (quartile 4) was significantly associated with cognitive impairment in models 1–3 (Model 1: OR 1.48; 95% CI, 1.02–2.14) and was abrogated only following the addition of energy-adjusted intakes of meat, fish, vegetables, fruits, and sodium (Model 4: OR 1.41; 95% CI, 0.97–2.06). The intake of tofu, miso soup, natto, and fermented soy foods was not associated with cognitive impairment in any of the models.

### Sex-stratified analyses

Of the 433 men, 189 were diagnosed with cognitive impairment at the mental health screening. The mean age and energy-adjusted genistein intake of men diagnosed with cognitive impairment was 74.7 (SD, 5.6) years, and 28.6 (SD, 13.1) mg/day, respectively.

Compared to men with the lowest energy-adjusted intake (quartile 1) of genistein in model 1, men with the highest intake (quartile 4) had significantly higher odds of cognitive impairment (OR 1.93; 95% CI, 1.06–3.52) (eTable 1). The association was abrogated in models 2–4 with non-significant associations throughout (Model 4: OR 1.68; 95% CI, 0.88–3.22). Compared to the lowest intake of fermented soy (quartile 1), the highest intake (quartile 4) of fermented soy was significantly associated with cognitive impairment in men in model 1 (OR 1.84; 95% CI, 1.03–3.29) and model 3 (OR 1.89; 95% CI, 1.02–3.47) but was ultimately non-significant in the fully adjusted model (Model 4: OR 1.94; 95% CI, 0.98–3.85). The intake of soy foods in general, tofu, miso, and natto was not associated with cognitive impairment in men.

Of the 603 women, 203 were diagnosed with cognitive impairment at the mental health screening. The mean age and energy-adjusted genistein intake of women diagnosed with cognitive impairment was 74.4 (SD, 5.6) years and 29.8 (SD, 18.6) mg/day, respectively. Compared to the lowest miso intake (quartile 1), those with high miso intake (quartile 3) had significantly lower odds of cognitive impairment (Model 1: OR 0.56; 95% CI, 0.33–0.95) (eTable 2). The association was only slightly attenuated by the addition of covariates and remained significant also in the fully adjusted model (Model 4: OR 0.54; 95% CI, 0.31–0.93). The intake of genistein, soy foods in general, tofu, natto, or fermented soy was not associated with cognitive impairment in women.

## DISCUSSION

This study has shown that individuals with the highest midlife intake (quartile 4) of the isoflavone genistein have an approximately 50% higher odds of late-life cognitive impairment compared to those with the lowest midlife intake (quartile 1).

Isoflavone and soy intake in Japan is among the highest in Asia,^28^ and our study has allowed us to study the association of long-term and high-dose midlife genistein and soy intake on late-life cognitive impairment. The major limitation of most observational studies investigating the association of soy and isoflavones on cognitive function is their concomitant assessment of both exposure and outcome. Individuals who suffer from cognitive impairment may not accurately recall their food intake or may have changed dietary habits as a direct consequence of their deteriorating health. This would be particularly important when assessing late-life participants, who may have a high prevalence of comorbidities. To the best of our knowledge, there is only one other observational study that has investigated midlife soy food intake, specifically tofu, on late-life cognitive function.^9^ Overall, our results are supported by previous studies, which suggest a detrimental effect of dietary soy intake on cognition.^7^^–^^9^ For overall soy intake, it was only in our final multivariable model where the highest soy intake became non-significantly associated with cognitive impairment. This abrogation suggests that the association between soy intake and cognitive impairment must be considered in the context of important dietary confounders; high vegetable and fruit intake may offset any risk of cognitive impairment, whereas sodium intake would be an important confounder with regards to cardiovascular risk.

In this study, we found no association between tofu intake and cognitive impairment. Hogervorst et al^7^ suggested that the detrimental effects of tofu on memory performance could be due to the use of formaldehyde as a preservative. If this indeed is the true cause of the association between tofu and cognitive function, even high tofu intake over many years would not constitute a health risk in Japan given that the use of formaldehyde as a preservative is prohibited. Additionally, in the study by White et al, the effect of midlife tofu intake explained only a fraction (2.3%) of the variance compared to the combined effects of age, education, and a history of stroke (27.8%).^9^ Consequently, the explanatory effect of tofu on cognitive impairment could be so small that it is already accounted for in our well-adjusted study.

One study has reported an inverse association between fermented soy products and cognitive impairment due to the higher concentration of folate,^29^ which is associated with a reduced risk of dementia.^30^ In contrast, in our main analysis, we found no association between midlife intake of fermented soy foods and late-life cognitive impairment. There are, however, two noteworthy results in our sex-stratified analyses: first, among men, intake of fermented soy in the highest quartile was significantly and positively associated with cognitive impairment in two of the models (Model 1 and Model 3). This suggests that the introduction of history of disease, in particular of stroke, in Model 3 results in negative confounding. Stroke is associated with a decline in cognitive function^31^ and increased risk of dementia.^32^ These significant associations were, however, abrogated when adjusting for important dietary confounders in the final model. Second, among women, there was an inverse association between the second highest quartile of midlife miso intake and late-life cognitive impairment. This finding is somewhat difficult to explain without speculation, in particular as the highest intake (quartile 4) of miso shows no association with the outcome. Moreover, the inverse association occurs only for one of the fermented soy products and not for the other, natto, or for the total intake of fermented soy. Taken together, this tells us that it is not a higher amount of fermented soy that is beneficial, which in turn is contrary to the effects expected with increased folate intake. Instead, one mechanism that could possibly help explain the results among women considers the estrogen-like properties of the isoflavones genistein and daidzein and their classification as phytoestrogens.^33^ Although estrogens may be neuroprotective and have beneficial effects on both learning and memory,^34^ high concentrations of phytoestrogens may have anti-estrogenic effects as they bind to the estrogen receptors and thus compete with endogenous estrogen.^35^ Moreover, genistein and daidzein are predominantly found in fermented soy foods,^36^ and daidzein has a biologically active metabolite, equol, which is produced by human intestinal bacteria.^37^ This could result in an increased bioavailability of isoflavones and consequently phytoestrogens in individuals who consume high amounts of fermented soy foods, as opposed to those who consume mostly non-fermented soy foods.

There are, to the best of our knowledge, only two studies that have investigated the long-term associations of isoflavone intake on cognitive impairment. These studies report either a protective effect only for women^10^ or no association,^11^ which are contrary to our own results. Our main findings instead show that high midlife intake of the isoflavone genistein is associated with cognitive impairment. Nevertheless, any recommendation to reduce the consumption of soy foods and isoflavones must be considered in the context of other studies, as well as the beneficial effects of isoflavones on cardiovascular health.^4^^–^^6^ In the present study, it was only the very highest level of genistein intake which was significantly associated with higher odds of cognitive impairment, indicating that only a very high consumption would have a detrimental effect. The overall high consumption of soy foods in Japan means that individuals who are in the top quartile of genistein consumption in this study would almost certainly be outliers in most other countries. Second, the effect of isoflavones must not necessarily be correlated with their consumed amount, as the inter-individual bioavailability of isoflavones may differ. In some individuals, daidzein is converted to equol by intestinal bacterial metabolism.^38^ It is suggested that those who are “equol producing” may constitute a sub-population with regards to the efficacy of soy foods on health outcomes.^38^ Indeed, one small cross-sectional Japanese study found that being an equol producer was a risk factor for MCI,^39^ which may support our findings. Future studies investigating the association between isoflavones and cognitive impairment should include and stratify participants on their equol-producing status. Third, the positive association between genistein and cognitive impairment was not seen in sex-stratified analyses. The direction of the associations in both men and women pointed towards increased odds of cognitive impairment with the highest genistein intake. However, the analyses also revealed that, among women, a “moderate” (quartile 2) or “high” intake (quartile 3) pointed towards an inverse association, albeit without any statistical significance. This suggests that larger study samples are required to reach sufficient statistical power and that the associations may be different between men and women. Future studies with sufficient sample sizes are required to investigate this further.

This study has a few limitations: we cannot exclude selection bias given that only approximately 15% of invited participants completed the mental health screening. We cannot with certainty state that participants were cognitively healthy at baseline; however, the likelihood of a cognitively impaired person completing the entire follow-up period with all follow-up surveys is very small. Generalization of results to other populations may be limited, as the dietary intake of soy food and genistein in Japan is substantially higher than in western countries. Moreover, although we have adjusted our analyses for age, we cannot exclude an age effect where individuals who consume a high amount of soy and isoflavone may live to a higher age where they develop cognitive impairment. We were also unable to test for equol-producing status, an important factor for the bioavailability of isoflavones, which may further limit generalizability. Although sex-stratified analyses were conducted, the results indicate that the strata may be too small to draw any wider conclusions based on the results. Future studies are encouraged to conduct sex-stratified analyses to further investigate the effect of midlife intake of phytoestrogens on cognitive impairment in women. Finally, due to the observational nature of the study, causality cannot be determined.

This study also has a number of considerable strengths: first, it is a study where genistein and soy intake was assessed at midlife, thereby minimizing any reverse causation bias. Second, the detailed questionnaire of the JPHC Study allows us to adjust for a large number of known confounders. Third, the soy and genistein intake was energy-adjusted and averaged over two assessments 5 years apart. This provides a stable and continuous exposure compared to using just a single time point. Finally, cognitive impairment was classified by a trained psychiatrist in accordance with DSM-IV-TR criteria.

### Conclusion

High midlife intake of the isoflavone genistein is associated with late-life cognitive impairment.

## SUPPLEMENTARY MATERIAL

eTable 1. Odds ratios and their confidence intervals of cognitive impairment in men according to energy-adjusted intake of isoflavone, soy food, tofu, miso soup, and fermented soy products

eTable 2. Odds ratios and their confidence intervals of cognitive impairment in women according to energy-adjusted intake of isoflavone, soy food, tofu, miso soup, and fermented soy products
