# Supplementary material for: Midlife Intakes of the Isoflavone Genistein and Soy and the Risk of Late-life Cognitive Impairment: The JPHC Saku Mental Health Study
Source: J Epidemiol. 2023 Jul 5;33(7):342–9. doi: 10.2188/jea.JE20210199 (PMC10257986; doi:10.2188/jea.JE20210199)
Supplement: Supplementary file 1 [file je-33-342-s001.pdf]

eTable 1. Odds ratios and their confidence intervals of cognitive impairment in men according to energy-adjusted intake of isoflavone, soy food, tofu, miso soup, and fermented soy products

	Quartile intake				
	**Quartile 1 (Low)**	**Quartile 2**	**Quartile 3**	**Quartile 4 (High)**	**P for trend**
**Isoflavone**					
Number (Events)	98 (34)	118 (52)	94 (43)	123 (60)	
mg/day, median [range]	13.8 [4.2–17.2]	21.1 [17.2–24.7]	28.7 [24.7–32.5]	40.7 [32.9–161.3]	
Model 1[a]					
OR (95% CI)	Reference	1.59 (0.90–2.81)	1.76 (0.95–3.27)	**1.93* (1.06–3.52)**	0.04
Model 2[b]					
OR (95% CI)	Reference	1.51 (0.84–2.70)	1.80 (0.95–3.40)	1.84 (0.99–3.41)	0.06
Model 3[c]					
OR (95% CI)	Reference	1.58 (0.87–2.87)	1.89 (0.99–3.62)	1.79 (0.95–3.36)	0.08
Model 4[d]					
OR (95% CI)	Reference	1.48 (0.81–2.72)	1.87 (0.97–3.62)	1.68 (0.88–3.22)	0.12
**Soy food**					
Number (Events)	108 (44)	93 (35)	105 (45)	127 (65)	
g/day, median [range]	37.4 [7.9–47.4]	55.8 [47.8–65.5]	76.3 [65.5–86.8]	114.1 [87.0–966.4]	
Model 1[a]					
OR (95% CI)	Reference	0.92 (0.51–1.67)	1.09 (0.61–1.94)	1.60 (0.90–2.84)	0.08
Model 2[b]					
OR (95% CI)	Reference	0.95 (0.52–1.74)	1.10 (0.61–2.00)	1.59 (0.88–2.87)	0.10
Model 3[c]					
OR (95% CI)	Reference	0.91 (0.49–1.69)	1.10 (0.60–2.03)	1.55 (0.85–2.83)	0.11
Model 4[d]					
OR (95% CI)	Reference	0.84 (0.45–1.58)	1.02 (0.55–1.90)	1.46 (0.80–2.69)	0.16

eTable 1 continued

**Tofu**					
Number (Events)	104 (43)	95 (38)	128 (61)	106 (47)	
g/day, median [range]	11.4 [–0.27 to 16.8]	23.1 [17.0–27.2]	33.5 [27.5–41.7]	56.8 [42.2–161.1]	
Model 1[a]					
OR (95% CI)	Reference	1.02 (0.57–1.82)	1.40 (0.81–2.41)	1.13 (0.64–2.00)	0.44
Model 2[b]					
OR (95% CI)	Reference	1.09 (0.60–1.99)	1.39 (0.80–2.42)	1.12 (0.62–2.01)	0.52
Model 3[c]					
OR (95% CI)	Reference	1.04 (0.57–1.91)	1.35 (0.77–2.36)	1.07 (0.59–1.94)	0.61
Model 4[d]					
OR (95% CI)	Reference	0.99 (0.53–1.83)	1.23 (0.69–2.18)	1.04 (0.56–1.92)	0.71
**Miso soup**					
Number (Events)	106 (43)	98 (35)	125 (57)	104 (54)	
mL/day, median [range]	38.5 [–0.1 to 76.2]	110.6 [77.1–135.8]	159.4 [139.6–187.9]	227.3 [189.6–639.2]	
Model 1[a]					
OR (95% CI)	Reference	0.75 (0.42–1.34)	1.26 (0.74–2.16)	1.55 (0.88–2.73)	0.05
Model 2[b]					
OR (95% CI)	Reference	0.75 (0.41–1.35)	1.24 (0.71–2.16)	1.50 (0.84–2.67)	0.07
Model 3[c]					
OR (95% CI)	Reference	0.83 (0.45–1.53)	1.30 (0.74–2.28)	1.67 (0.92–3.02)	0.04
Model 4[d]					
OR (95% CI)	Reference	0.85 (0.45–1.59)	1.26 (0.69–2.29)	1.73 (0.89–3.37)	0.06

eTable 1 continued

**Natto**					
Number (Events)	116 (49)	97 (40)	109 (45)	111 (55)	
g/day, median [range]	3.4 [–0.36 to 5.5]	7.6 [5.6–9.4]	12.3 [9.5–16.2]	24.6 [16.2–83.1]	
Model 1[a]					
OR (95% CI)	Reference	1.00 (0.57–1.76)	0.97 (0.56–1.71)	1.37 (0.78–2.40)	0.32
Model 2[b]					
OR (95% CI)	Reference	1.02 (0.57–1.81)	0.97 (0.55–1.72)	1.41 (0.79–2.53)	0.30
Model 3[c]					
OR (95% CI)	Reference	1.02 (0.57–1.83)	0.98 (0.55–1.74)	1.35 (0.75–2.43)	0.38
Model 4[d]					
OR (95% CI)	Reference	1.02 (0.56–1.85)	0.96 (0.54–1.74)	1.37 (0.75–2.51)	0.37
**Fermented soy (Natto+Miso)**					
Number (Events)	98 (36)	100 (40)	129 (58)	106 (55)	
g/day, median [range]	48.3 [10.2–86.0]	119.2 [86.6–146.7]	171.2 [146.8–199.3]	240.1 [200.0–647.5]	
Model 1[a]					
OR (95% CI)	Reference	1.07 (0.59–1.93)	1.45 (0.83–2.51)	**1.84* (1.03–3.29)**	0.02
Model 2[b]					
OR (95% CI)	Reference	1.00 (0.55–1.82)	1.40 (0.79–2.46)	1.73 (0.95–3.14)	0.04
Model 3[c]					
OR (95% CI)	Reference	1.07 (0.58–1.98)	1.45 (0.81–2.59)	**1.89* (1.02–3.47)**	0.02
Model 4[d]					
OR (95% CI)	Reference	1.10 (0.59–2.08)	1.40 (0.76–2.58)	1.98 (1.00–3.93)	0.04

CI, confidence interval; OR, odds ratio.

*p<0.05
[a]Model 1 is adjusted for age, education and energy-adjusted folate intake;
[b]Model 2 is additionally adjusted for alcohol consumption, smoking, body mass index, and physical activity;
[c]Model 3 is additionally adjusted for history of diabetes mellitus and use of prescribed medications;
[d]Model 4 is additionally adjusted for energy-adjusted intake of fish, meat, vegetables, fruits, and sodium.

e**Table 2.** Odds ratios and their confidence intervals of cognitive impairment in women according to energy-adjusted intake of isoflavone, soy food, tofu, miso soup, and fermented soy products

	Quartile intake				
	**Quartile 1 (Low)**	**Quartile 2**	**Quartile 3**	**Quartile 4 (High)**	**P for trend**
**Isoflavone**					
Number (Events)	150 (50)	148 (43)	141 (43)	164 (67)	
mg/day, median [range]	13.7 [5.9–17.2]	21.0 [17.3–24.8]	28.3 [24.8–32.6]	40.0 [32.6–146.1]	
Model 1[a]					
OR (95% CI)	Reference	0.78 (0.47–1.30)	0.88 (0.52–1.48)	1.40 (0.85–2.30)	0.13
Model 2[b]					
OR (95% CI)	Reference	0.78 (0.47–1.29)	0.87 (0.52–1.47)	1.39 (0.84–2.29)	0.14
Model 3[c]					
OR (95% CI)	Reference	0.78 (0.47–1.30)	0.88 (0.52–1.49)	1.39 (0.84–2.29)	0.14
Model 4[d]					
OR (95% CI)	Reference	0.78 (0.47–1.31)	0.83 (0.49–1.41)	1.35 (0.81–2.24)	0.21
**Soy food**					
Number (Events)	152 (53)	132 (32)	145 (43)	174 (75)	
g/day, median [range]	39.0 [16.2–50.9]	59.6 [50.9–70.1]	79.8 [70.2–92.9]	118.2 [93.0–798.2]	
Model 1[a]					
OR (95% CI)	Reference	0.60 (0.35–1.03)	0.77 (0.46–1.28)	1.42 (0.87–2.29)	0.08
Model 2[b]					
OR (95% CI)	Reference	0.61 (0.36–1.04)	0.77 (0.46–1.28)	1.42 (0.87–2.31)	0.08
Model 3[c]					
OR (95% CI)	Reference	0.62 (0.36–1.06)	0.76 (0.45–1.26)	1.43 (0.88–2.32)	0.09
Model 4[d]					
OR (95% CI)	Reference	0.60 (0.35–1.04)	0.72 (0.43–1.21)	1.37 (0.84–2.25)	0.13

eTable 2 continued

**Tofu**					
Number (Events)	144 (43)	152 (44)	159 (63)	148 (53)	
g/day, median [range]	11.2 [0.31–15.9]	20.7 [15.9–26.0]	31.6 [26.1–39.7]	54.8 [40.0–186.9]	
Model 1[a]					
OR (95% CI)	Reference	0.91 (0.55–1.51)	1.43 (0.87–2.34)	1.15 (0.70–1.90)	0.27
Model 2[b]					
OR (95% CI)	Reference	0.91 (0.55–1.52)	1.46 (0.89–2.39)	1.17 (0.70–1.93)	0.24
Model 3[c]					
OR (95% CI)	Reference	0.90 (0.54–1.51)	1.47 (0.89–2.41)	1.16 (0.70–1.93)	0.24
Model 4[d]					
OR (95% CI)	Reference	0.92 (0.55–1.55)	1.47 (0.89–2.44)	1.17 (0.69–1.96)	0.25
**Miso soup**					
Number (Events)	155 (57)	147 (47)	125 (33)	176 (66)	
mL/day, median [range]	34.1 [–0.12 to 58.4]	79.6 [58.7–104.8]	129.0 [105.8–143.8]	181.5 [144.8–421.9]	
Model 1[a]					
OR (95% CI)	Reference	0.81 (0.50–1.32)	**0.56* (0.33–0.95)**	0.97 (0.61–1.54)	0.73
Model 2[b]					
OR (95% CI)	Reference	0.82 (0.50–1.34)	**0.54* (0.32–0.93)**	0.96 (0.60–1.52)	0.65
Model 3[c]					
OR (95% CI)	Reference	0.81 (0.49–1.32)	**0.54* (0.32–0.92)**	0.96 (0.61–1.53)	0.67
Model 4[d]					
OR (95% CI)	Reference	0.78 (0.47–1.29)	**0.54* (0.31–0.93)**	0.97 (0.60–1.59)	0.74

eTable 2 continued

**Natto**					
Number (Events)	141 (48)	157 (51)	138 (44)	167 (60)	
g/day, median [range]	3.9 [–0.46 to 6.3]	8.6 [6.3–10.7]	14.4 [10.8–18.3]	23.7 [18.4–72.9]	
Model 1[a]					
OR (95% CI)	Reference	0.87 (0.53–1.43)	0.87 (0.52–1.46)	1.04 (0.64–1.70)	0.82
Model 2[b]					
OR (95% CI)	Reference	0.84 (0.51–1.39)	0.85 (0.51–1.43)	1.01 (0.62–1.65)	0.89
Model 3[c]					
OR (95% CI)	Reference	0.82 (0.49–1.35)	0.84 (0.50–1.41)	1.03 (0.63–1.69)	0.81
Model 4[d]					
OR (95% CI)	Reference	0.82 (0.49–1.36)	0.81 (0.48–1.37)	1.00 (0.61–1.64)	0.96
**Fermented soy (Natto+Miso)**					
Number (Events)	147 (47)	155 (55)	131 (38)	170 (63)	
g/day, median [range]	46.5 [3.6–70.2]	90.2 [70.3–119.4]	144.3 [120.0–163.0]	193.2 [163.2–440.7]	
Model 1[a]					
OR (95% CI)	Reference	1.18 (0.73–1.92)	0.81 (0.48–1.36)	1.17 (0.73–1.89)	0.83
Model 2[b]					
OR (95% CI)	Reference	1.17 (0.71–1.91)	0.78 (0.46–1.32)	1.13 (0.70–1.84)	0.94
Model 3[c]					
OR (95% CI)	Reference	1.16 (0.71–1.90)	0.78 (0.46–1.32)	1.14 (0.70–1.85)	0.91
Model 4[d]					
OR (95% CI)	Reference	1.09 (0.66–1.79)	0.79 (0.46–1.36)	1.12 (0.67–1.87)	0.88

CI, confidence interval; OR, odds ratio.
*p<0.05
[a]Model 1 is adjusted for age, education and energy-adjusted folate intake;
[b]Model 2 is additionally adjusted for alcohol consumption, smoking, body mass index, and physical activity;
[c]Model 3 is additionally adjusted for history of diabetes mellitus, and use of prescribed medications;
[d]Model 4 is additionally adjusted for energy-adjusted intake of fish, meat, vegetables, fruits, and sodium.
